# What Do You Need to Know before Studying Chagas Disease? A Beginner’s Guide

**DOI:** 10.3390/tropicalmed8070360

**Published:** 2023-07-10

**Authors:** José A. De Fuentes-Vicente, Nancy G. Santos-Hernández, Christian Ruiz-Castillejos, Eduardo E. Espinoza-Medinilla, A. Laura Flores-Villegas, Mariana de Alba-Alvarado, Margarita Cabrera-Bravo, Adriana Moreno-Rodríguez, Dolores G. Vidal-López

**Affiliations:** 1 Instituto de Ciencias Biológicas, Universidad de Ciencias y Artes de Chiapas, Tuxtla Gutiérrez 29039, Mexico; 2 Facultad de Medicina, Universidad Nacional Autónoma de México, Ciudad de México 04510, Mexico; 3 Facultad de Ciencias Químicas, Universidad Autónoma Benito Juárez de Oaxaca, Oaxaca 68120, Mexico

**Keywords:** Chagas disease, guide, *Trypanosoma cruzi*, triatomine, reservoirs

## Abstract

Chagas disease is one of the most important tropical infections in the world and mainly affects poor people. The causative agent is the hemoflagellate protozoan *Trypanosoma cruzi*, which circulates among insect vectors and mammals throughout the Americas. A large body of research on Chagas disease has shown the complexity of this zoonosis, and controlling it remains a challenge for public health systems. Although knowledge of Chagas disease has advanced greatly, there are still many gaps, and it is necessary to continue generating basic and applied research to create more effective control strategies. The aim of this review is to provide up-to-date information on the components of Chagas disease and highlight current trends in research. We hope that this review will be a starting point for beginners and facilitate the search for more specific information.

## 1. Introduction

Vector-borne diseases are a complex set of diseases caused by pathogens that are transmitted to humans by a living vector, usually an arthropod [1]. According to estimates by the World Health Organization (WHO), vector-borne diseases account for more than 17% of all infectious diseases and cause more than 700,000 deaths per year [2].

Some of the most important vector-borne diseases are Chagas disease, malaria, dengue, yellow fever, leishmaniasis, and onchocerciasis. Although most of these diseases have a cosmopolitan distribution, a major burden is concentrated in tropical and subtropical areas; this is in large part due to ideal environmental conditions for vectors, though demographic, cultural, and socioeconomic factors are also involved [3]. Most of these vectors are blood-sucking insects, such as mosquitoes, kissing bugs, fleas, flies, and ticks, which ingest the pathogen while feeding on an infected host and then transmit it to another host where disease may develop.

**Figure 1:**
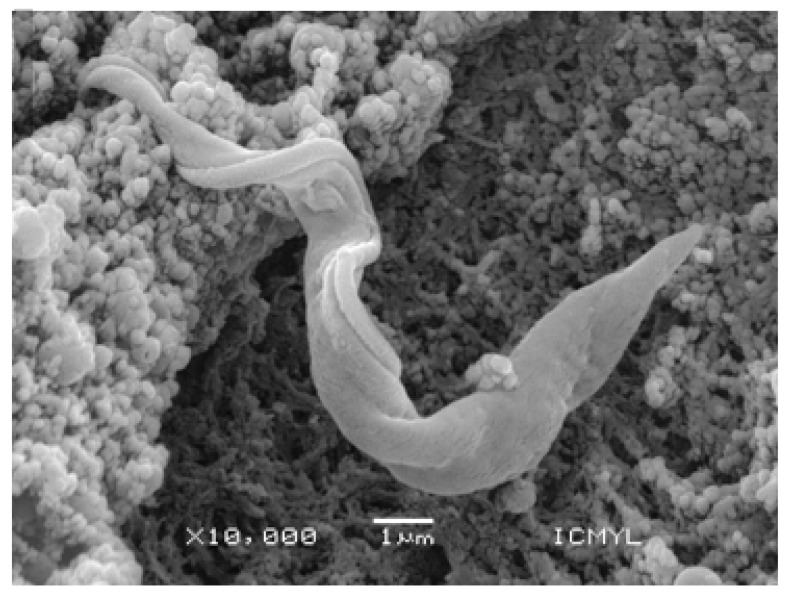
The *Trypanosoma cruzi* parasite (in the intestine of the vector) is the causative agent of Chagas disease (scanning slectron microscope).

**Figure 2:**
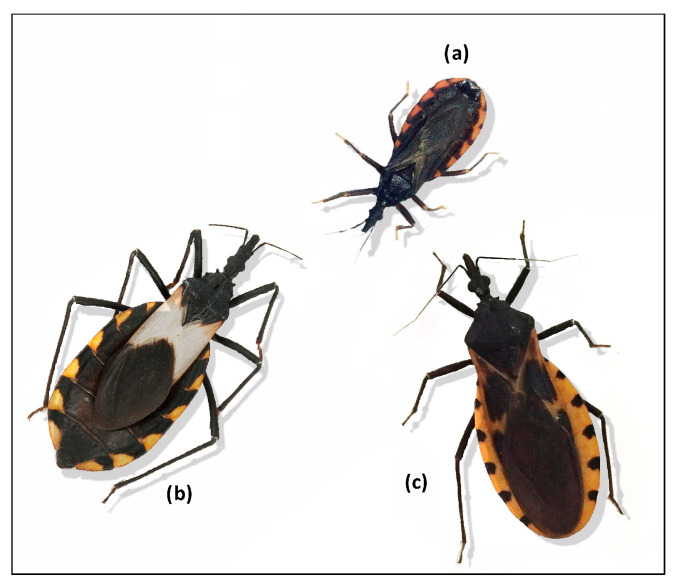
Kissing bug specimens, vectors of *Trypanosoma cruzi*: (**a**) *Triatoma barberi*, (**b**) *T. pallidipenis,* and (**c**) *T. imidiate*.

American trypanosomiasis, also known as Chagas disease, is caused by the protozoan *Trypanosoma cruzi* (Figure 1). This parasite is transmitted through the feces or urine of triatomine insects known as kissing bugs (Figure 2), and according to the WHO, it is one of the most important neglected tropical infections in the world [4]. Although this disease is addressed by public health policies in several Latin American countries, control initiatives have not been as successful as expected, largely due to the complexity of the disease. Knowledge about the components of Chagas disease has grown greatly over the past two decades, but there are still many unknowns that must be resolved in order to establish truly effective strategies. For example, we have little understanding of how genetic and morphological variation among vectors influences their ability to colonize or reinfest homes, become resistant to insecticides, and transmit *T. cruzi*. Furthermore, new studies are making it ever clearer that there is wide genetic diversity in the parasite itself, which leads to different clinical manifestations of the disease in humans and hinders the development of an effective drug [5]. Therefore, new lines of research are being conducted, and new generations of young scientists could hold the key to improving our understanding and changing paradigms surrounding Chagas disease.

In light of these developments, this paper is intended to be an entry point for beginning scholars and non-experts on Chagas disease. We will provide general, up-to-date (though not exhaustive) information on the main components of this disease and orient the reader towards more specific sources of information for their study purposes.

## 2. A Brief History of Chagas Disease and the Current Situation

While conducting a campaign against malaria in Brazil in 1908, Carlos Chagas analyzed the rectal contents of a kissing bug and observed flagellated organisms [6]. Subsequently, he sent some insects to his mentor, Oswaldo Cruz, and performed inoculations on experimental animals to reproduce, isolate, and identify the parasite, which he named *T. cruzi* in honor of his mentor. In addition, he detected a similar trypanosome in the blood of a 2-year-old girl (Berenice) who presented fever, hepatomegaly, and splenomegaly, thus establishing the first human case of the disease [6]. Today, experimental inoculations in animal models are still used to evaluate the virulence of different strains and attempt to predict clinical complications of infection in endemic regions, e.g., [7,8], where parasitological parameters such as the prepatent period, parasitemia, mortality, and cell tropism are evaluated (for a review of these parameters, see De Fuentes-Vicente et al. [9]).

**Figure 3:**
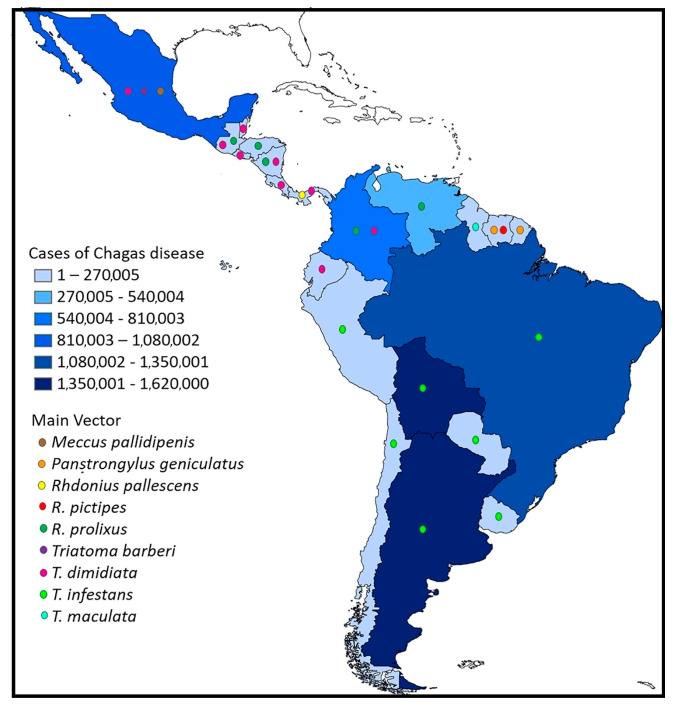
Distribution of Chagas disease and the main triatomine vectors [15].

Chagas disease is widely distributed in 21 countries in the Americas (Figure 3) and currently affects between 6 and 7 million people worldwide [10]. The fact that this number has decreased from ~18 million people in previous decades is evidence of the effectiveness of chemical vector control campaigns [11]. Although the insect vector does not occur in Europe, the migration of infected people is causing serious problems, and the number of infected people outside the endemic range of the disease is increasing [12,13]. Therefore, it is important to address this problem from a multidimensional perspective under the migration approach [14].

Two drugs are currently available to treat Chagas disease—benznidazole and nifurtimox—but the general consensus is that their efficacy is limited to early infections and young people [16]. However, other factors, such as the strain of the parasite and the host’s immune response, may influence the drugs’ activity and efficacy. The mechanisms of action of both drugs, as well as the biodistribution and potential new formulations of benznidazole, have been reviewed by Sales-Junior et al. [16], where the importance of creating new therapies for the disease is also emphasized. In this sense, new formulations are being studied as treatment alternatives (e.g., [17]); at the same time, some drugs that are already approved to treat other diseases are being investigated as potential treatments for Chagas disease through structure-based drug repositioning [18].

The costs of treatment, together with the inability to work due to the symptoms of Chagas disease, represent a considerable economic loss globally, particularly for endemic countries [19]. Mathematical modeling shows the economic advantages of preventing disease [20] and treating infected people early on [21,22], which is undoubtedly beneficial in severely affected countries with poor economies.

## 3. Life Cycle of *Trypanosoma cruzi*

**Figure 4:**
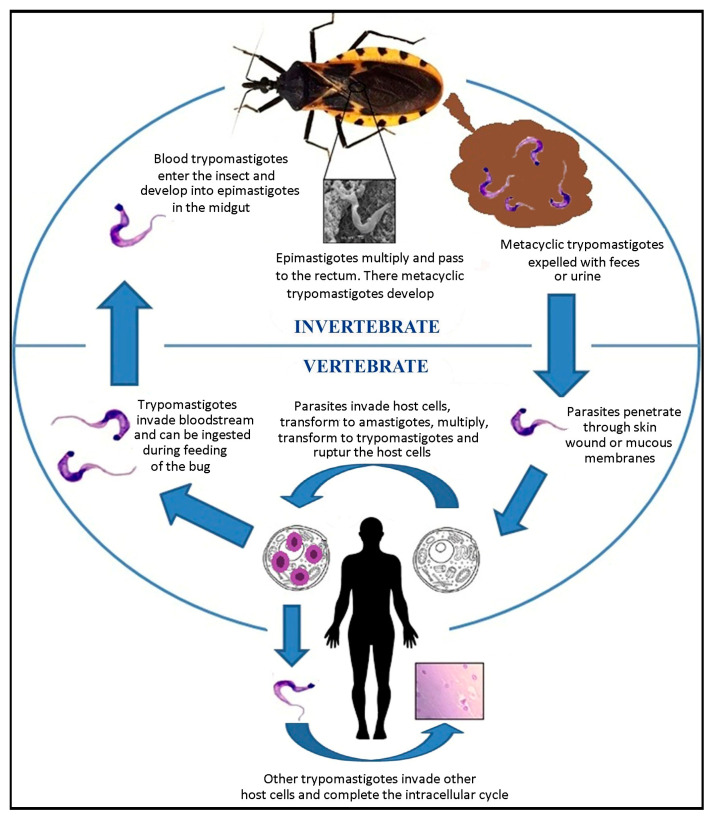
Life cycle of *Trypanosoma cruzi* (modified from [15]).

The life cycle of *T. cruzi* is well known (see [23]) and consists of several morphologically distinct developmental stages—the metacyclic trypomastigote, blood trypomastigote, amastigote, and epimastigote [24]. An overview is shown in Figure 4. When the metacyclic forms enter humans, proteins such as gp82 assist in Ca^2+^ mobilization, which facilitates cell invasion [25]. After approximately nine cycles of intracellular division, the parasites lyse the cell, invade other cells, and create nests of amastigotes in the host’s tissues. When parasites occur in the bloodstream, they can be absorbed by a triatomine during feeding and infect it for life. In the perimicrovillar membrane of the insect midgut, the parasites transform into epimastigotes and replicate by binary fission. They then migrate to the rectal ampulla, where they will become metacyclic trypomastigotes, which are expelled along with the feces and can infect another host to repeat the cycle [23]. In the rectum of the vector, *T. cruzi* glycoproteins play a key role in membrane attachment [26] and potentially in strain virulence.

The life cycle of *T. cruzi* is more complex than it appears and is influenced by biotic and abiotic factors that impact the epidemiology of the disease (reviewed by De Fuentes Vicente et al. [15]). For example, seasonality, parasite strain, vector species, and vector nutrition play key roles in replication within the vector. In the vertebrate host, altitude, parasite genetic structure, and host immune response influence the parasite-host relationship [27,28].

Although the vectorial route is the main transmission mechanism in the *T. cruzi* cycle, other routes of transmission include blood transfusion, organ transplantation, sexual [29], transplacental [30], and oral transmission, as well as the handling of mammalian carcasses [31], each with different degrees of severity and importance in endemic countries. An in-depth review of the challenges in controlling these infection mechanisms has been conducted by Shikanai-Yasuda [32].

## 4. Generalities of *Trypanosoma cruzi*

*T. cruzi* is a eukaryotic organism belonging to the order Kinetoplastida, a group of unicellular organisms possessing extranuclear DNA. This extra portion of genetic material, known as the kinetoplast, is located at a specific point in the mitochondrium. It represents almost 20% of the parasite’s total genetic material and is mainly involved in the processing of mitochondrial messenger RNAs [33]. Although it appears to be a relatively simple eukaryotic cell, the biochemistry of *T. cruzi* reveals a truly complex organism (see [34,35]). Notwithstanding, like other parasites, *T. cruzi* has lost the ability to synthesize some essential molecules and has to take them from its vertebrate host during parasitism (e.g., sialic acid [36]). These molecules are therefore studied as potential therapeutic targets due to their role as virulence factors (e.g., trans-sialidases [37]; calreticulin [38]).

The surface molecules of *T. cruzi* vary according to the evolutionary form but are mostly composed of mucin-type glycoproteins involved in protection and infectivity processes. Trans-sialidases, cruzipains, and others are also present in smaller amounts (for a complete review of the main molecules, see Pech-Canul et al. [39]). Comparative proteomic studies of different *T. cruzi* strains show significant differences in cell surface molecule composition, which appears to be closely related to the immunopathogenesis of the disease. Therefore, unraveling the biological importance of these molecules will contribute to a better understanding of the parasite-host interaction and the design of more effective drugs [40].

Given its transmission mechanism, *T. cruzi* is cataloged in the stercoraria group, which refers to parasites transmitted through the feces of their vectors. In contrast, *T. rangeli*, the non-pathogenic sister trypanosome of *T. cruzi,* is transmitted through the saliva of the same vectors during the bite [41]. Interestingly, *T. cruzi* does not survive in the vector’s hemocele like *T. rangeli* because the insect’s immune system manages to eliminate it [42]. Therefore, the microhabitat of *T. cruzi* in triatomines is restricted to the digestive tract, where it interacts with the insect’s natural microbiota [43]. This interaction has led to a very interesting and promising line of research that attempts to understand the interaction and competition of the parasite with the microbiota as a strategy to control Chagas disease [44]. For example, the bacterium *Serratia marcenses* produces a pigment called prodigiosin, which alters the mitochondrial function of the parasites, eventually causing their elimination [45].

In parallel, the use of genetically modified symbionts, known as paratransgenesis, has been gaining ground in recent years. The modified symbionts must be ingested by the vectors and are expected to express molecules that interfere with pathogen replication and transmission [46]. To this end, it is important to first identify the natural symbionts of the vectors to determine potential candidates, as has already been performed with the vector *Triatoma dimidiata* [47]. However, one obstacle to paratransgenesis is that *T. cruzi* can induce changes in the insect microbiota that have been associated with a decreased immune response against the parasite [48]. In this sense, paratransgenesis for the control of Chagas disease should take into account the parasite-vector interaction, particularly in terms of the resulting behavioral and physiological changes.

*T. cruzi* is easily recognizable under light microscopy by the characteristic C- and S-shaped appearance of the blood trypomastigotes and the big kinetoplast in stained blood smears. The trypomastigote form measures approximately 20 µm long and 1 µm wide, while the epimastigote is slightly smaller [23]. The amastigote is rounded in shape with a diameter of 5 µm and no flagellum [24]. The morphological differences among the life cycle stages are accompanied by internal changes. For example, the nuclear shape is altered, the mitochondrial DNA is rearranged, and the volume of the kinetoplast is modified. One of the most fascinating works on morphological and structural changes during *T. cruzi* cell differentiation was performed by Gonçalves et al. [24], where intermediate forms are identified that may be useful for future biochemical and molecular work.

## 5. Origin and Genetic Variation of *Trypanosoma cruzi*

Thanks to advances in molecular techniques, there has been great progress in the last two decades in understanding the origin and evolution of *T. cruzi*, as well as its evolutionary relationships with other trypanosome species. Although several evolutionary models have been proposed, quite a few questions remain. One early hypothesis suggests *T. cruzi* originated when South America, Antarctica, and Australia separated from Africa, an idea known as the “southern supercontinent hypothesis” [49,50]. According to this hypothesis, trypanosomes evolved in isolation in early South American land mammals. However, the relatively low trypanosome diversity in South American mammals and the presence of sister trypanosomes in Africa and Australia contradict the hypothesis that *T. cruzi* originated and evolved in geographic isolation [51].

An alternative hypothesis, the “bat seeding hypothesis”, has been gaining ground among the scientific community, mainly based on molecular evidence suggesting that *T. cruzi* evolved from a broader clade of bat-restricted trypanosomes from Africa as a result of five independent switches from bats to terrestrial mammals, where the hematophagous vector played an important role. Strong evidence for this is the close genetic similarity of *T. cruzi* to *T. marinkellei*, a bat trypanosome [50]. Some models indicate a divergence of the *T. cruzi* ancestor from *T. marinkellei* approximately five to seven million years ago, and several introgression events gave rise to the genetic variants of *T. cruzi* [52].

The genetic variation of *T. cruzi* is unquestionable, and its classification has been gradually changing. The wide genetic variation is partly due to clonal population structure, genome plasticity, and an abundance of repeated sequences [53], but it is also due to the diversity of hosts and vector species, which each exert different selective pressures on *T. cruzi* [54]. Interestingly, genetic exchange in *T. cruzi* appears to occur, but only infrequently [5]. In the 1990s, mini-exon gene and random amplified polymorphic DNA (RAPD) analysis revealed two major divergent phylogenetic lineages: TcI and TcII [55].

**Figure 5:**
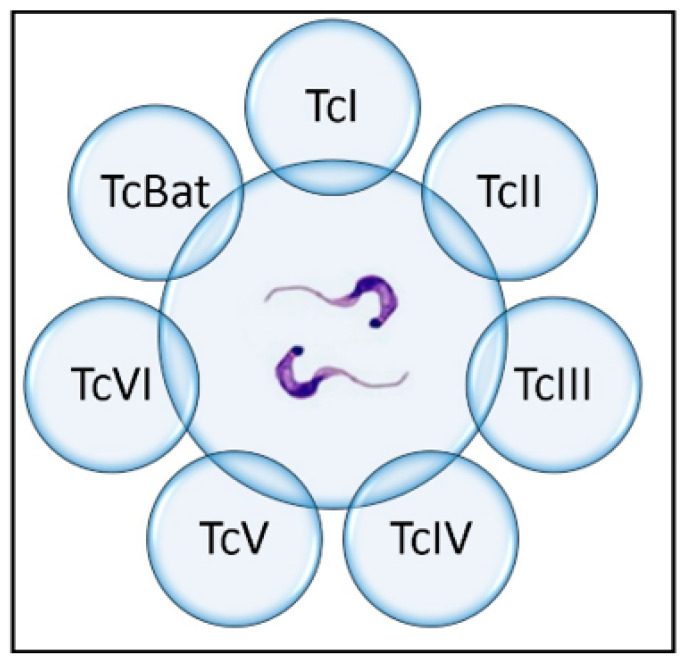
Genetic classification of *Trypanosoma cruzi*.

Subsequently, wide intragroup genetic heterogeneity was described within TcII, so it was subdivided into five subgroups: TcII a–e [56]. Subsequent scientific consensus and the use of new molecular tools then led to the decision to reclassify the parasite into seven discrete typing units (DTUs)—TcI–TcVI and Tcbat [57], which persists to this day (Figure 5). This classification is expected to advance knowledge of the population structure of *T. cruzi*, the evolution of the different groups, and the definition of epidemiological characteristics [58].

## 6. Vector Insects (Hemiptera, Reduviidae, and Triatominae)

The Triatominae are a well-characterized subfamily of the family Reduviidae (Hemiptera), although their phylogenetic origin (monophyletic, polyphyletic, or paraphyletic) remains controversial [59]. Within this family, the triatomines (subfamily Triatominae) are the only mainly hematophagous group. Triatomines’ mouthparts are adapted to this feeding type, allowing them to pierce the skin of vertebrates, and the molecules contained in the saliva are more benign than those of other predatory reduviids [54]. An in-depth analysis of the evolution of the hematophagous habit in Triatominae has been carried out by Otalora-Luna et al. [59], which evidences all of the behavioral and physiological changes of this group to adapt to hematophagy. This evolutionary event gave triatomines marked anthropological importance since it included them in the life cycle and transmission of the *T. cruzi* parasite [59]. Although blood is indispensable for the development of triatomines, they are able to exploit other food sources as a survival mechanism in environments with a low density of food sources [60]. Blood is rich in protein but lacking in vitamins; therefore, triatomines maintain a close relationship with their microbiota, which provides them with the nutrients absent in their diet [61]. The absence of this microbiota can alter the correct development of the insect [62]. Naturally, the microbiota can be transmitted to offspring through coprophagy [43].

**Figure 6:**
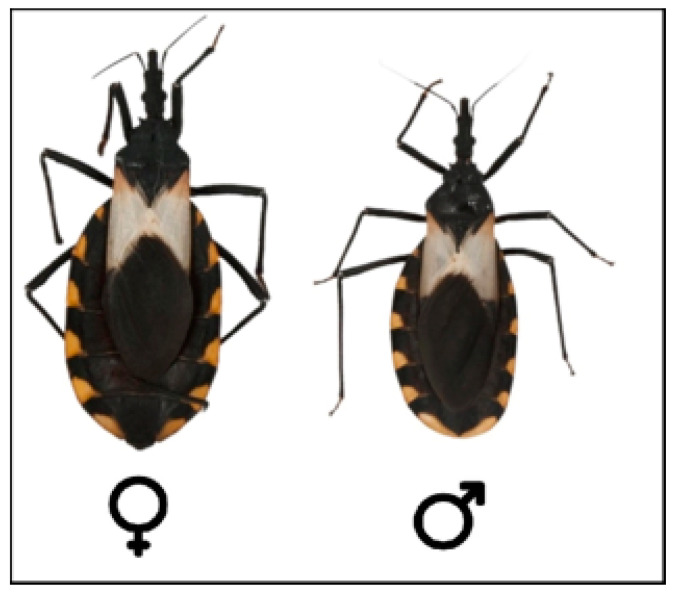
Female and male specimens of *T. pallidipenis*. Note the differences in the posterior border.

Triatomine development is hemimetabolous since the nymphs present similar biological characteristics to those of adults. The life cycle comprises an egg stage followed by five nymphal stages before reaching the adult stage. In adults, the sexes can be differentiated because the posterior border in males is continuous, while in females it is interrupted by the ovipositor apparatus (Figure 6) [63]. Triatomine species vary widely in size, ranging from 5 mm in *Alberprosenia goyovargasi* from Venezuela to 45 mm in *Dipetalogaster maxima* from Mexico [64]. The life cycle of triatomines and their phenotype in general can be altered by biotic and abiotic factors, including the presence of *T. cruzi*. However, the effects of the trypanosomatid seem to depend on the respective combinations with strains/species of triatomines [15].

Triatomine eggs have variable morphology, ranging from spherical to cylindrical, and they have an operculum through which the nymph emerges. Egg analysis has taxonomic value because it provides a reliable classification of species [65]. The incubation time of the egg is 10 to 30 days, after which the soft, pink nymphs emerge and are ready to feed by the second or third day after emergence [66]. Females mate two to three days after becoming adults. Although they do so several times, only one copulation is necessary to provide sperm, which is stored in the spermatheca for subsequent egg-laying. During the average life of the female, she can lay 100 to 200 eggs, depending on her nutritional status and the species [64]. However, infection with *T. cruzi* might alter reproductive performance [67].

## 7. Distribution and Richness of Triatomines

**Figure 7:**
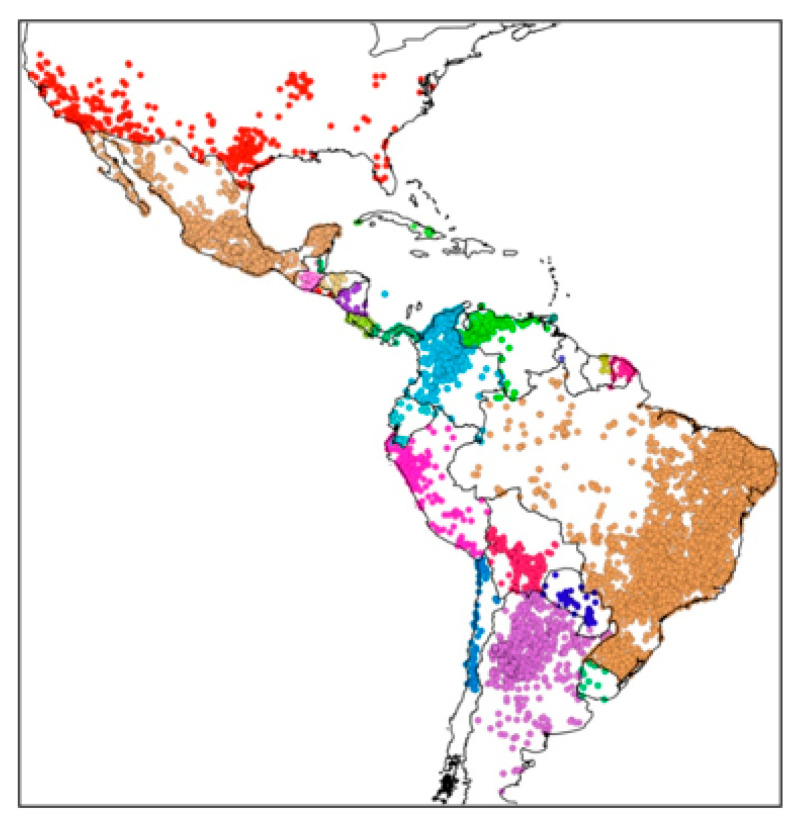
Geographic distribution of triatomines based on reports of their occurrence in America. Each color represents the triatomines of a country (made by Ceccarreli et al. [69]).

Triatomines are distributed throughout the Americas, from the southern United States to northern Argentina, presenting a gradual increase in species richness from the poles towards the equator [68]. One of the most up-to-date and comprehensive works on the distribution of triatomines based on presence records is that of Ceccarelli et al. [69] (Figure 7). However, due to the wild habitat of most species, there are still many unexplored areas, and it is likely that new species will remain to be described.

The subfamily Triatominae is constantly changing, and new taxonomic information is continuously presented to clarify its phylogeny. Perhaps the greatest challenge in triatomine classification is the lack of a unifying species concept [70]. Within this group of insects, *T. dimidiata* is by far the most taxonomically complex group, and there are still many questions about its classification [71]. Overall, the current classification of Triatominae recognizes 157 species grouped into five tribes (Alberproseniini, Bolboderini, Cavernicolini, Rhodniini, and Triatomini) and 18 genera [72]. Two tribes are considered to have epidemiological importance in the transmission of *T. cruzi*: the Rhodniini and the Triatomini.

The tribe Rhodniini Pinto, 1926, includes two genera: *Psammolestes* Bergroth, 1911, and *Rhodnius* Stal, 1859. The three species corresponding to the genus *Psammolestes* are 11–15 mm long with truncated heads. They are associated with stick nests woven by birds of the *Dendrocolaptinae* or *Furnariidae* subfamilies. They have a variable distribution, ranging from the plains of Colombia and Venezuela to the Gran Chaco, the Argentine Pampas, and Brazil [70]. *Rhodnius* species average 15–20 mm in length. Twenty-one species have been described and are distributed from Central America to the Southern Cone. They are associated with palm trees, bird nests, and domestic and peridomestic habitats [73]. They have been extensively studied due to their epidemiological importance and geographic distribution and classified into three groups (pictipes, pallescens, and prolixus) based on their geographic distribution and morphology [74]. The genera *Psammolestes* and *Rhodnius* are different in morphology and ecological habits, but both are arboreal and exhibit ecological adaptations to tree canopies (*Rhodnius*) and bird nests (*Psammolestes*) [73].

Tribe Triatomini Jeannel, 1919, consists of seven genera: *Dipetalogaster* Usinger, 1939; *Eratyrus* Stal, 1859; *Hermanlentia* [64]; *Linshcosteus* Distant, 1904; *Panstrongylus* Latreille, 1811; *Paratriatoma* Barber, 1938; and *Triatoma* Laporte, 1832. It has a wide geographic distribution, encompassing a variety of ecotopes, although most species appear to be naturally associated with rupicolous (rock-breeding) habitats. According to their geographic distribution, they are grouped into three lineages: (1) Triatoma of Central or North America (plus Old World species), which contains *Dipetalogaster*, *Eratyrus*, *Linshcosteus*, *Paratriatoma*, and *Panstrongylus*; (2) Triatoma of South America; and (3) the *dispar* complex, which is distributed from the Andes in Venezuela through Bolivia [73].

## 8. Overview of the Ecology of Triatomine Bugs

Triatomine insects can inhabit a wide variety of ecological environments, from very generalized to highly specialized, including the domestic ecotope where vectorial infection in humans occurs [75]. Only a few species of triatomines have adapted to living inside homes, most notably *T. dimidiata*, *R. prolixus*, and *T. infestans*. In this sense, human dwellings have become the “microhabitat” of intradomiciliary populations, and the conditions are less hostile than the external environment. Reasonably, these species are targeted by vector control campaigns in endemic areas, with good results [76]. However, insecticide resistance, as well as re-infestation of the dwelling with the same or other species, remains a major barrier to elimination. Still, other species may inhabit the periphery of dwellings (peridomicile), where they feed on blood from farm animals and occasionally enter the home to feed on humans [77]. The mechanisms underlying the ability to adapt to a domestic or peridomestic ecotope are not entirely clear, but genetic variation and phenotypic plasticity undoubtedly play an important role, as is the case with *T. dimidiata* [78]. Understanding the ability of triatomine species to occupy wild, peridomestic, or domestic habitats is essential to optimizing current control strategies and anticipating future epidemiological trends [79].

Wild triatomines mainly occupy vertebrate nests and burrows, where temperature and humidity play a key role in their ecology and are related to differences in population abundance (for a detailed review, see Justi and Galvão [80]). With global warming, high temperatures and relatively low humidity are expected to occur and influence these insects’ life histories and geographic distribution [81]. Physiologically, increased temperature can accelerate the vector’s developmental cycle, and in low humidity conditions, the cycle is shortened by metabolic alterations and dehydration (e.g., [82]). Likewise, the distribution of triatomines is related to temperature, and under the effects of climate change, they are expected to extend their distribution towards more temperate areas [83], although this may depend on the thermal tolerance of each species. For example, *R. prolixus* is expected to expand into new areas, while *T. infestans* is expected to reduce its geographic distribution [84]. In the case of wild species such as *Mepraia spinolai* and *M. gajardoi*, their expansion will occur in areas with similar climatic conditions, the former in semi-arid areas and the latter in coastal areas with an arid climate in Chile [85]. Species’ future expansion is also influenced by infection with *T. cruzi*; some distribution models suggest a relationship between the niche used by the insects and the presence of the parasite [86].

Triatomine insects use a variety of sensory mechanisms to communicate with each other and to locate their hosts during the night, when feeding activity is most intense. This nocturnal behavior probably serves as a strategy to ensure sufficient time to feed while mammals sleep, since feeding can take up to 15–20 min. Some sensory modalities to locate their hosts consist of the detection of carbon dioxide gradients, heat, smell, humidity, and air flows [87]. Their thermal sense is considered to be one of their most sensitive systems and perhaps the most important in locating their food source. However, in the case of exothermic hosts with low temperatures, they may be able to use other chemical signals, such as recognition of amines and short-chain aliphatic acids, aldehydes, alcohols, and lactic acid (see Barrozo et al. [88] for a more in-depth review). As expected, infection with *T. cruzi* also plays a key role in the sensory ecology of triatomines. An analysis of the antennal phenotype of wild individuals demonstrated significant differences in the antennal sensilla between infected and uninfected individuals [89]. It is likely that during the process of coevolution between *T. cruzi* and triatomines, the parasite induced an adaptive manipulation in the vector phenotype to facilitate its transmission, but there is little information available on this topic to date, so our understanding is still limited.

## 9. *T. cruzi* Reservoirs

The parasite-reservoir relationship is a complex biological system in which one organism (the parasite) lives and reproduces inside another individual (the reservoir), allowing it to continue to circulate in nature. In order to persist, a parasite must not kill its reservoir but, at the same time, produce pathogenesis that allows it to continue its life cycle [11,90]. More than 180 mammalian species have been identified as acting as *T. cruzi* reservoirs. This wide range of reservoirs further increases the complexity of the parasite and allows it to exploit different environments throughout the Americas. Infection in wild animals can occur through the feces of the vector, as occurs with humans, or through predation by the insect vectors or infected animals [90]. Although the transplacental route may be an important part of the wild cycle, this mode of transmission has only been studied in a few animal models [91].

The main reservoir species found to be naturally infected with *T. cruzi* are found within seven orders: Marsupialia, Rodentia, Carnivora, Primata, Chiroptera, Lagomorpha, and Artiodactyla, as well as the superorder Xenarthra [92]. In particular, opossums (*Didelphis* spp.) are considered to be among the most important wild reservoirs in the *T. cruzi* cycle. Interestingly, the parasite goes through an extracellular cycle in the opossum scent glands and can infect other mammals by contamination, similar to insect vectors [93]. Another important aspect of opossums is the close relationship they seem to have with the DTU TcI, which suggests they have undergone many coevolutionary scenarios. This has led to the proposal of the hypothesis that early forms of *T. cruzi* were associated with opossums and subsequently passed to triatomine insects that fed on opossum blood [54].

Most *T. cruzi* reservoirs inhabit wild environments; however, domestic animals, farm animals, and synanthropic animals (e.g., rodents) can act as links that connect the wild cycle with the peridomestic and domestic cycles, hence the importance of monitoring this cycle in areas close to human settlements (e.g., [94]). In addition, with monitoring studies, it is possible to identify mammals that can act as parasite amplifiers or bioindicators (sentinels) in areas where parasite transmission to humans is frequent.

One current paradigm surrounding *T. cruzi* reservoirs is that non-mammal vertebrates, such as birds, amphibians, and reptiles, are refractory to the parasite due to their body temperature (39–41°), antigen incompatibility, lack of recognition, and cell signaling. Nevertheless, these animals represent a primary food source for insect vectors in wild environments. Recently, the presence of *T. cruzi* DNA in different organs was identified in an owl species (*Tito furcata*) [95], suggesting a change in the current paradigm is necessary. Similarly, experimental infection with *T. cruzi* has also been demonstrated in lizards, but their role as potential natural reservoirs is not entirely clear [96]. On the other hand, fish are not considered potential reservoirs of *T. cruzi* due, logically, to their living conditions. Notwithstanding, experimental infection has been successfully performed in zebrafish (*Dania rerio*) for use as an in vivo model to study parasite motility in the vascular system [97].

A group that has gained growing interest in recent years has been bats. Several studies have confirmed natural infection in populations throughout the Americas (e.g., [94,98]). Their ability to fly long distances and the synanthropic status of some species suggest great importance in the wild cycle and in the dispersal of the parasite. For example, infected bats could serve as links to the domestic cycle because they are eaten by cats in urban areas [99]. In addition, the presence of *T. cruzi* DNA has been documented in the salivary glands of hematophagous bats from Peru, suggesting a potential transmission of the parasite through bites [100].

## 10. Clinical Forms and Diagnosis of Chagas Disease

**Figure 8:**
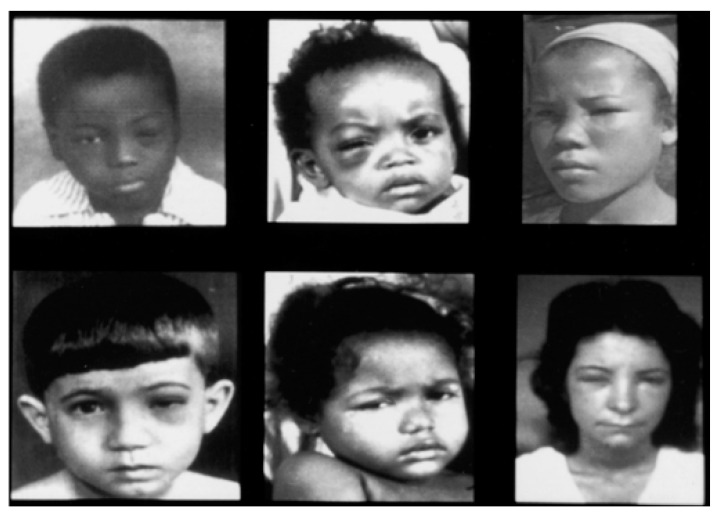
Patients with Chagas disease have the Romaña sign, which is characteristic when the parasite enters through the conjunctiva (Prata [102]).

Currently, two distinct phases of Chagas disease in humans are recognized: the acute and chronic stages. The acute stage begins within days of infection and is characterized by the parasite circulating and replicating significantly in the bloodstream (parasitemia) [101]. Most acute cases of Chagas disease are asymptomatic, or the symptoms are very mild. In symptomatic patients, headache, dizziness, fever, hepatomegaly, and splenomegaly are the most frequent symptoms, which disappear spontaneously after a few days or weeks [102]. When infection occurs through the conjunctiva, unilateral palpebral edema in both the upper and lower eyelids, known as Romaña’s sign, occurs (Figure 8). The fact that an estimated 80% of patients present as asymptomatic patients and the appearance of symptoms that are common in other diseases (mainly headache, dizziness, and fever) make early suspicion and diagnosis of the disease difficult. In addition, little or no access to healthcare systems in affected populations makes diagnosis even more difficult [103].

The chronic stage of the disease begins when the number of parasites decreases significantly in the bloodstream, approximately 4 to 8 weeks after infection [101]. Therefore, the infection remains clinically silent while the parasite slowly replicates in tissues and creates nests of amastigotes. In 70% of cases, infected patients may have no clinical manifestations for the rest of their lives. However, in the remaining 30%, clinical complications may occur 10–30 years after infection. These complications are mainly related to cardiac, digestive, or neurological alterations, which can lead to death [104]. Sadly, many of the diagnoses of Chagas disease are made when the evolution of the disease has caused irreversible damage and there is little to be done.

The tools for the diagnosis of Chagas disease depend largely on the stage of the disease in the patient. During the acute stage, direct observation of blood under a 40× microscope or by the microhematocrit method are relatively inexpensive options to detect the parasite. A polymerase chain reaction (PCR) test or IgM-type antibody detection may also be a viable, albeit more expensive, option [105].

In the chronic stage of the disease, serological tests for the detection of IgG antibodies are the most reliable tool, with a sensitivity of at least 99%. According to the WHO, the use of two different serological tests is recommended for the correct diagnosis of Chagas disease [10]. At this stage, the use of the PCR technique has generated controversy because, in untreated infected individuals, this technique gives a positive result in only 40–60% of cases [106]. An exhaustive and up-to-date review of the accuracy of diagnostic tests was conducted by Candía-Puma et al. [107], including a meta-analysis of literature findings.

For the diagnosis of complications arising from *T. cruzi* infection, the electrocardiogram is the main method of detecting alterations or abnormalities in heart function. Chest X-rays can also help reveal cardiomegaly or signs of heart failure. For suspected alterations in the digestive system, an esophagram can be used to determine dilatation of the viscera (megaesophagus or megacolon) (see Balouz et al. [105]).

As mentioned above, Nifurtimox, launched by Bayer in 1967, and Benznidazole, launched by Roche in 1971, are the only drugs approved for the treatment of Chagas disease. However, many patients present adverse reactions to these drugs, to the point that they may even suspend treatment. Furthermore, they should not be administered to pregnant women, people with kidney or liver failure, or those with a history of neurological or psychiatric disorders [10]. In light of this, there is a great need to change these treatment regimens (see Morillo and Echeverria [108]) or to formulate new, more tolerable antiparasitics without sacrificing efficacy.

## 11. Immunopathogenesis of Chagas Disease

The pathogenicity of *T. cruzi* is regulated by several genetic, immunological, and environmental factors related to both the vertebrate host and the parasite. The most relevant are the genetic polymorphisms of the host and the antigen constitution described for various strains of *T. cruzi*. When *T. cruzi* enters the mammalian host, the innate immune response is activated by the recognition of various membrane proteins. Although the complement system is one of the main defense pathways, *T. cruzi* has proteins, such as CRIT, CRP, and GP58/68, that are able to inhibit C3 and thereby prevent the anchoring of the membrane attack complex (MAC). In this way, the parasite prevents the activation of all complement pathways and is able to establish itself within the host [109,110].

One of the most important immune response cells for the control of parasitemia in the acute stage are macrophages, which recognize parasite surface GLP glycoproteins, such as Tc52, via toll-like receptors (TLR2, TLR3). These receptors are expressed on different cells, such as NK cells, granulocytes, neutrophils, and eosinophils. When a parasite is recognized by a macrophage, it is phagocytosed and enters the phagolysosome to be exposed to reactive oxygen species and destroyed. However, *T. cruzi* has peroxidase and superoxide dismutase enzymes, allowing it to survive by evading the inducible nitric oxide synthase pathway within the macrophage. Although some parasites can be destroyed, evasion of this innate immune response is frequently successful and allows the parasite to continue its invasion of other cells (not erythrocytes and thrombocytes) [111]. In fact, due to this ability, *T. cruzi* also promotes its cellular internalization by generating an “eat me” signal similar to the signal emitted by cells undergoing apoptosis [112].

Similarly, to enter host cells, *T. cruzi* synthesizes trans-sialidases in order to sequester sialic acid from the surface of host cells. By surrounding itself with sialic acid, the parasite can enter the cell via the vesicle signaling pathway. Once inside the cell cytoplasm, the parasite forms a parasitophorous vacuole from which it will exit and begin the process of binary fission in the cytoplasm. This process occurs in all nucleated cells to which *T. cruzi* has tropism, such as myocardiocytes, neurons, and smooth muscle cells [113]. In addition, after activation of the innate immune response, APC cells will have the capacity to present parasitic antigens through the major histocompatibility complex, a process by which CD4+ and CD8+ cells will be activated. When the lymphocytes are activated by means of their TCR, co-stimulatory signals, and cytokines, they can activate their effector or memory function.

Depending on the cytokine microenvironment, various Th lymphocyte responses can be defined. A proinflammatory Th1-type response orchestrated by IFN has generally been described in intracellular parasites such as *T. cruzi*. The presence of this response activates phagocytosis and NK cells at the onset of infection and is critical for parasite control. However, when a deregulation of this response occurs, a generation of proinflammatory cytokines is observed during the chronic phase [114,115]. The production of TNF-α continues during the chronic phase and generates inflammatory foci with tissue damage, ultimately generating fibrosis, which evolves into cardiomyopathy. Likewise, in this phase, it has been observed that patients express a marked pattern of Th1-/Th17-type cytokines associated with their ECO and Electrocardiographic manifestations [116].

Tissue tropism of *T. cruzi* has been related to the genetic constitution of the parasite strain [58]. For example, TcI *T. cruzi* strains have a marked cardiotropism and tend to generate a response characterized by inflammatory cellular infiltration and lesions, such as the degeneration of cardiac fibers and fibrosis [116]. Meanwhile, other strains attack the digestive system and tend to reduce or destroy the neurons of the intramural nerve plexuses, leading to the elongation, dilation, and hypertrophy of a portion of the colon, leading to a clinical picture of chronic constipation [117]. Likewise, the esophagus can be affected (chagasic megaesophagus), which is manifested by the inability of the esophageal sphincter to relax in response to swallowing and the absence of peristalsis in the esophageal body [118]. Among the disorders that are potentially caused by *T. cruzi*, chagasic cardiomyopathy is the most severe and is associated with high mortality in Latin America [119].

The pathogenic process of Chagas cardiomyopathy develops through different mechanisms caused by the intracellular multiplication of the amastigote phase in the heart, which leads to lysis of cardiac cells and activation of the specific immune response against the parasite. Four pathogenic mechanisms involved in the formation of fibrotic lesions in the myocardium of infected patients are currently recognized (For a detailed review, see Bonney and Engman [120]).

Myocytolysis is caused by the presence and replication of the parasite in cardiac cells, which results in cardiac cell lysis, generating chronic inflammatory processes and myocarditis.

Neuronal parasitism occurs when *T. cruzi* invades intramural neurons, resulting in cardiac conduction disorders and arrhythmias.

Increased endothelin concentrations are caused by the inflammatory process. It can induce thrombosis, tissue hypoxia, and ultimately necrosis.

Autoimmune processes may result from molecular mimicry between proteins of the myocardium and *T. cruzi*. For example, myosin (the main protein of cardiac muscle) is similar to the B13 protein of the parasite. Some of the possible mechanisms for the development of autoimmunity may be the release of host cell components, the generation of autoantibodies, and reactive T cells. These autoantibodies generate lesions through the complement pathway and macrophage activation [121].

At present, Chagas disease continues to be a challenge for scientific research from several perspectives. The study of the immunopathology of lesions in Chagas disease, inflammation, and the cells involved in this process, as well as the predisposition of certain strains of *T. cruzi* for myocardium, continues to be studied [122], which, together with the regulation of the immune response by the parasite and the host as a delicate balance between them, could define the course of the disease towards chronicity in the vertebrate host [123].

## 12. Final Comments

More than 110 years after its discovery, Chagas disease continues to be a matter of great global concern due to its distribution and prevalence. From our academic trenches, our best weapon is to continue generating new knowledge that will allow us to better understand its dynamics. Likewise, it is important to strengthen ties with the government and the health sector to generate joint strategies and try to prevent unfavorable scenarios. On the other hand, it is necessary to create awareness in society about this public health problem and motivate their participation to prevent future infections.

With this review, we hope the reader has gained a better understanding of the complexity of the disease and a more comprehensive view of its components from the information provided. The challenges of Chagas disease are still great, and it is necessary to develop research under different approaches since the approach from a single perspective (social, biological, medical, etc.) can greatly delimit the scope of research.

## Data Availability

Not applicable.
